# Response of Depression to Botulinum Toxin Treatment: Agitation as a Predictor

**DOI:** 10.3389/fpsyt.2015.00055

**Published:** 2015-04-20

**Authors:** Roumen Milev

**Affiliations:** ^1^Department of Psychiatry, Queen’s University, Kingston, ON, Canada

**Keywords:** major depressive disorder, botulinum toxin, prediction, treatment, agitation

Major depressive disorder (MDD) is a mental disorder, characterized by low mood, lack of interest or pleasure, and a cluster of other vegetative and cognitive symptoms, occurring for a period of time and causing significant distress or impairment of functioning. It has a relapsing and recurring course, sometimes becomes chronic. It is a common disorder with a life time prevalence of 16.6% (1) and 12 months prevalence of 6.7% (2). MDD affects patients’ functioning and is associated with significant personal and societal burden.

It was predicted that MDD would rank second by disability adjusted life years (DALY) among all disorders worldwide in year 2020, contributing with 5.7% of DALY and second only to ischemic heart disease (3).

The episodes of depression in MDD, if left untreated, may last for months or years. There are many treatments available for depression including psychotherapy (4), pharmacotherapy (5), neurostimulation (6), and other complementary and alternative medicine methods (7), as summarized in the CANMAT clinical practice guidelines. Although there are numerous treatments available, the results are far from ideal, with significant proportion of patients not achieving remission and continuing to suffer with symptoms of the disorder and functional impairment. Therefore, the quest for new and better treatment approaches is continuing.

There is a small but growing body of evidence suggesting that botulinum toxin may be useful for treatment of depression. Botulinum toxin is a protein produced by *Clostridium Botulinum* and is highly toxic and lethal. In medical use, when injected, it induces strong flaccid muscle paralysis, and this effect is used in the treatment of a variety of different disorders characterized by muscle over activity and spasticity and in hyperhidrosis. More recently, botulinum toxin use has skyrocketed in cosmetic medicine, where it leads to significant visual improvement of wrinkles, due to the paralysis of the underlying muscles.

The main hypothesis for the proposed beneficial effects of botulinum toxin in depression is through the facial feedback. Low mood and depression are often associated with sad facial expression. It has long been observed that MDD patients often have deep vertical frown lines and different signs, such as “omega melancholicum” sign and Veraguth’s folds, have been described (8). Injecting botulinum toxin and improving facial expression would lead to improvement of symptoms of depression.

In an open study of botulinum toxin injected into the frown muscles of 10 depressed patients, Finzi and Wasserman (9) reported that 8 of the 10 patients went into remission after one treatment of botulinum toxin. This observation was followed by a small randomized, double blind, controlled trial by Wollmer et al. (10), who examined 30 patients randomly assigned to receive injection of botulinum toxin or saline placebo. The patients were mainly women and the authors reported response rate [as measured by 50% improvement on Hamilton Depression Rating Scale (HAM-D)] of 60% in the active group vs 13.3% in the placebo group. Of interest, the presence of significant vertical frown line was required in order to be included in this study. Soon after, a larger randomized controlled trial was reported by Finzi and Rosenthal (11), which included 85 patients. This study used Montgomery–Asberg Depression Rating Scale (MADRS) as the primary outcome measure and did not require a pronounced vertical frown for inclusion in the study, but the results were very similar to the study by Wollmer. One issue discussed in both randomized and controlled trials was the fact that most patients guessed correctly whether they were in the placebo group or in the botulinum toxin active group, which might have also influenced their response rates.

In the current *post hoc* analysis (12) of their original study (10), Wollmer et al. are trying to determine predictors for better response to botulinum toxin in patients with MDD. After conducting further analysis of the data, they found that high baseline agitation (as measured on item 9 of HAM-D) is the main predictor of response to botulinum toxin with sensitivity of 100%, specificity of 56%, and overall precision of 87%. Agitation may be associated with more dynamic motor activity, leading to more changes in the facial expression. The baseline appearance of the glabellar frown line was not a predictor in this analysis.

All the limitations of this study (e.g., small size, *post hoc* analysis, etc.) are known and clearly stated. Nevertheless, this type of analysis brings an important clinical point: we not only need to know which treatment works, but also who are the patients that would likely benefit most! The authors are to be commended on this fine attempt to answer that.

## Conflict of Interest Statement

The author declares that the research was conducted in the absence of any commercial or financial relationships that could be construed as a potential conflict of interest.
